# Supplementary material for: Orchestration of protein acetylation as a toggle for cellular defense and virus replication
Source: Nat Commun. 2018 Nov 23;9:4967. doi: 10.1038/s41467-018-07179-w (PMC6251895; doi:10.1038/s41467-018-07179-w)
Supplement: Supplementary file 1 — Supplementary Information [file 41467_2018_7179_MOESM1_ESM.pdf]

Orchestration of protein acetylation as a toggle for cellular defense and virus replication

Murray, et al.

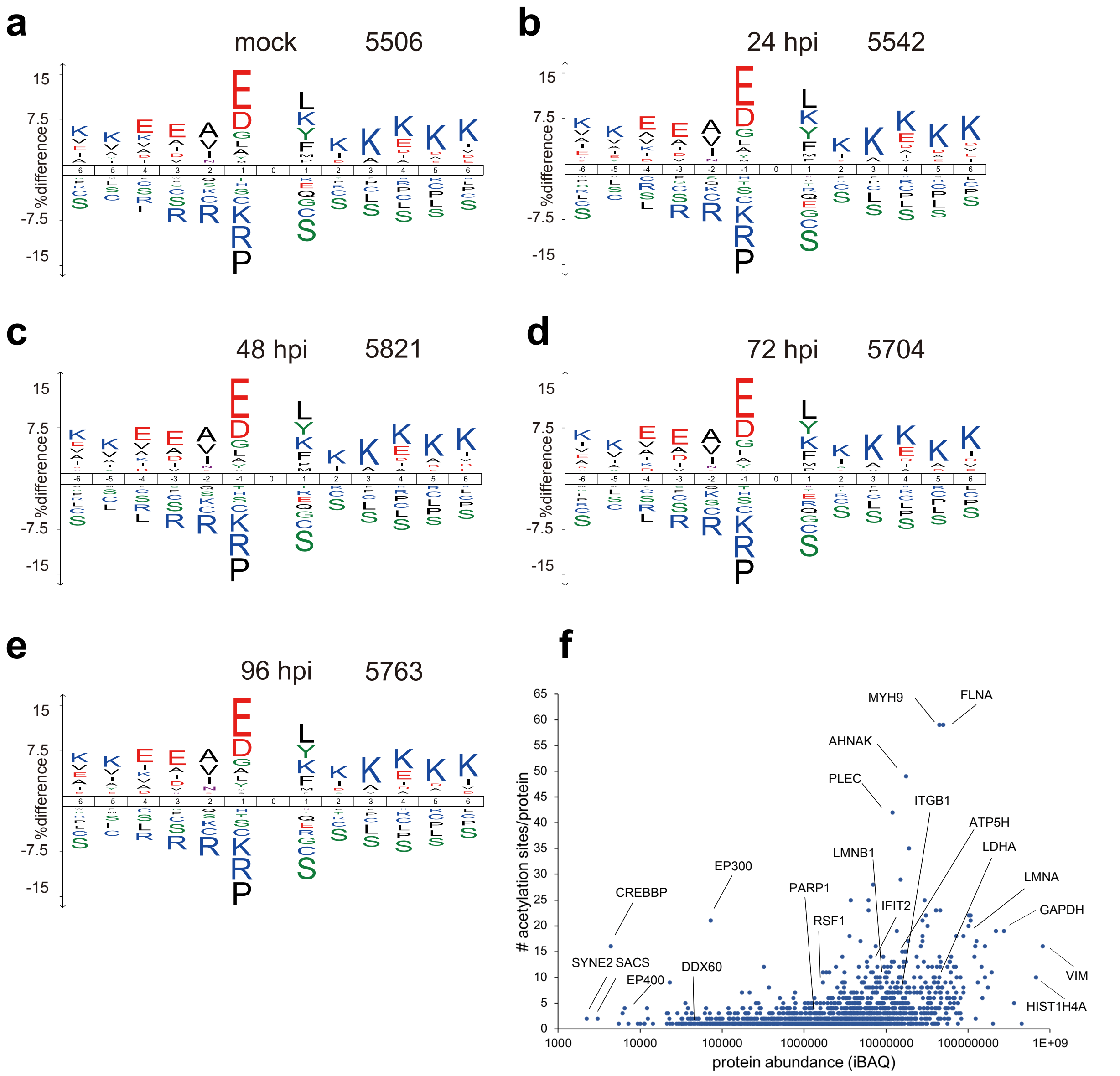

Supplementary Figure 1. Analysis of acetylated cellular peptides from uninfected and infected cells. Motifs from **a** uninfected and infected cells at **b** 24 hpi, **c** 48 hpi, **d** 72 hpi, and **e** 96 hpi were analyzed by IceLogo. Data represent results from a percentage difference scoring method with a P-value cut-off equal to 0.01 for the six amino acids flanking the acetylated lysine. The height of the letter at each position represents the difference in the amino acid residue frequency between the experimental set and the reference set. Numbers on the top indicate the number of peptides used for generating IceLogos. **f** Relationship between number of acetylation

sites and corresponding protein abundance. Protein quantification was performed using the iBAQ algorithm.

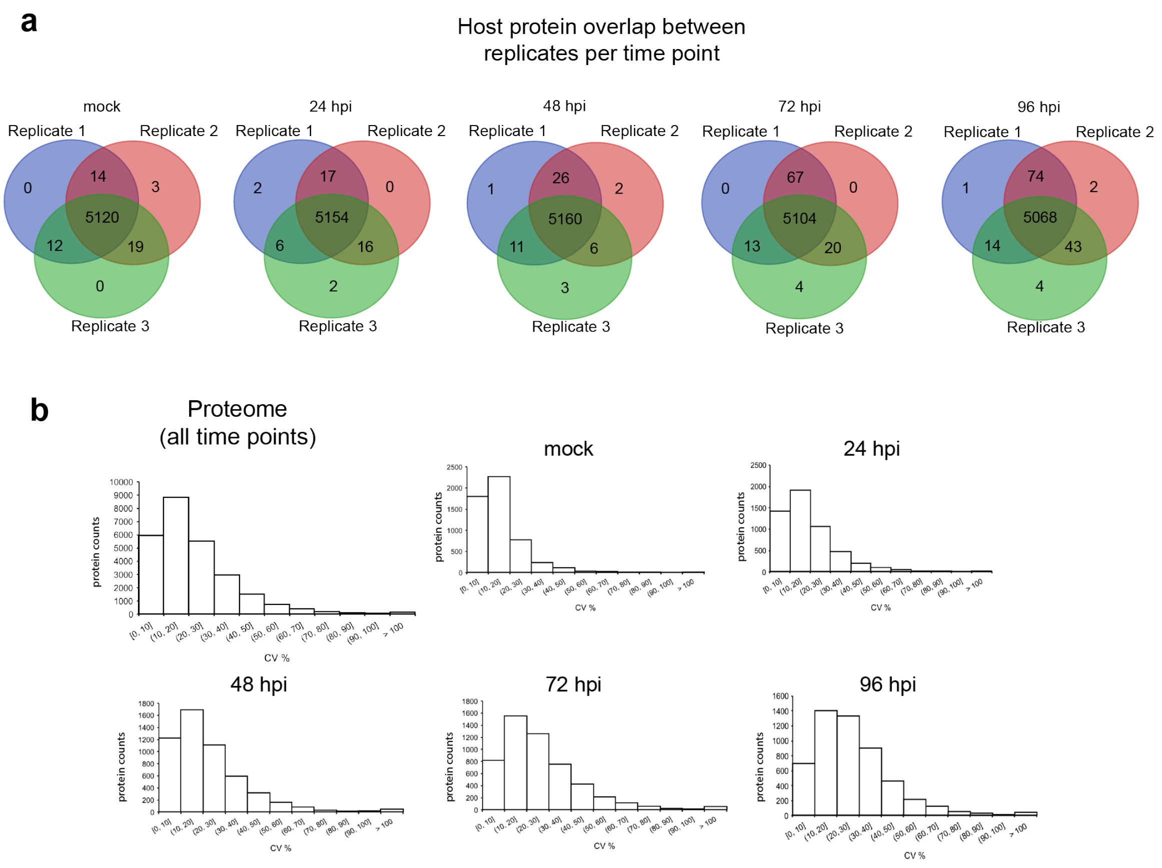

c

Replicate	mock rep1	mock rep2	mock rep3	24 hpi rep1	24 hpi rep2	24 hpi rep3	48 hpi rep1	48 hpi rep2	48 hpi rep3	72 hpi rep1	72 hpi rep2	72 hpi rep3	96 hpi rep1	96 hpi rep2	96 hpi rep3
# proteins	5146	5156	5151	5179	5187	5178	5198	5194	5180	5184	5191	5141	5157	5187	5129
# peptides detected	45438	48270	47449	47040	48908	47798	47383	48943	47381	46934	48476	46169	44233	48311	45768
# peptides quantified	43618	46188	45532	45098	46777	45746	45454	46803	45319	45015	46500	44068	42327	46273	43663

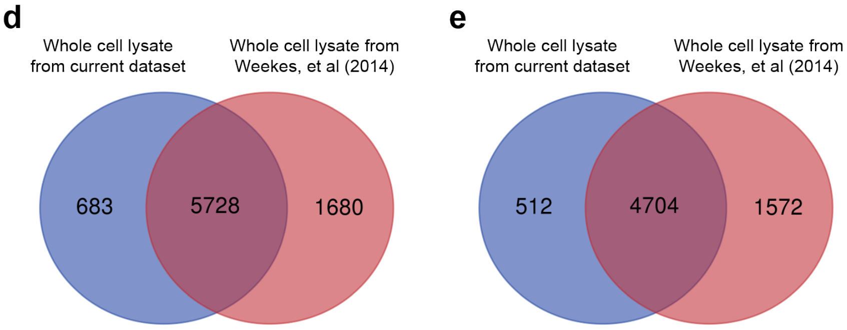

Supplementary Figure 2. Comparison of the proteome datasets from the biological replicates, as well as with prior literature. a Venn diagrams showing the overlap in host proteins quantified between three biological replicates at each time point during infection. **b** Distribution of the coefficient of variation (CV %) of all time points combined, and individual

time points for host whole proteome. **c** Comparison of number of host proteins and number of corresponding detected and quantified unmodified peptides across all three biological replicates in all time points. **d, e** Comparison of the number of identified proteins from the current proteome dataset and a previous study (Weekes, et al 2014). **d** Number of identified proteins with more one or more unique peptides (not including isoforms). **e** Number of identified proteins with two or more unique peptides (not including isoforms).

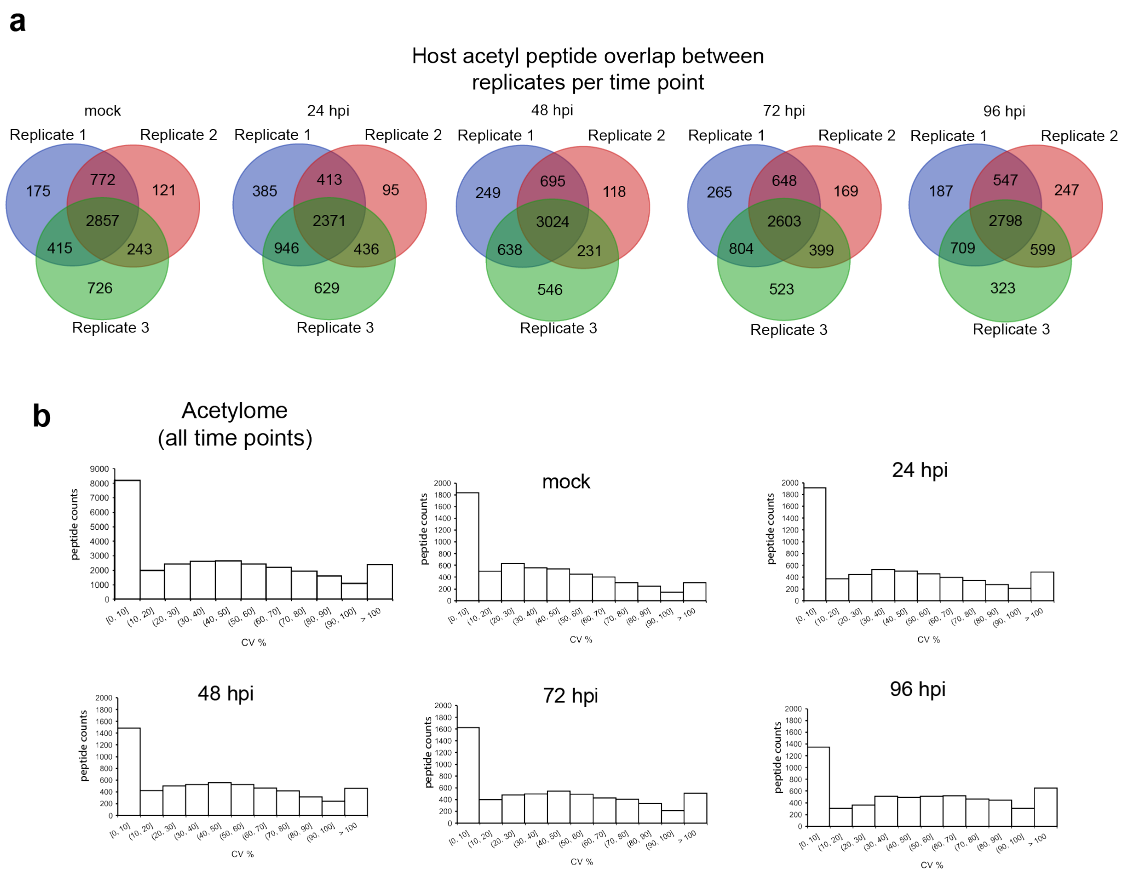

Supplementary Figure 3. Comparison of the acetylome datasets from the biological replicates. a Venn diagrams showing the overlap in host acetylated peptides filtered and used in the quantification analysis between three biological replicates at each time point during infection. **b** Distribution of the coefficient of variation (CV %) of all time points combined, and individual time points for host peptides in the acetylome. CV percentages do not include imputed values.

a

Replicate	mock rep1	mock rep2	mock rep3	24 hpi rep1	24 hpi rep2	24 hpi rep3	48 hpi rep1	48 hpi rep2	48 hpi rep3	72 hpi rep1	72 hpi rep2	72 hpi rep3	96 hpi rep1	96 hpi rep2	96 hpi rep3
# acetylated peptides	4219	3993	4241	4115	3315	4382	4606	4068	4439	4320	3819	4329	4241	4191	4429
# acetylated proteins	1608	1563	1604	1606	1367	1631	1692	1559	1639	1610	1502	1593	1566	1590	1602
% imputed acetyl peptide abundance (only used for trend analysis)	27	31	26	29	42	24	20	29	23	25	34	25	26	27	23

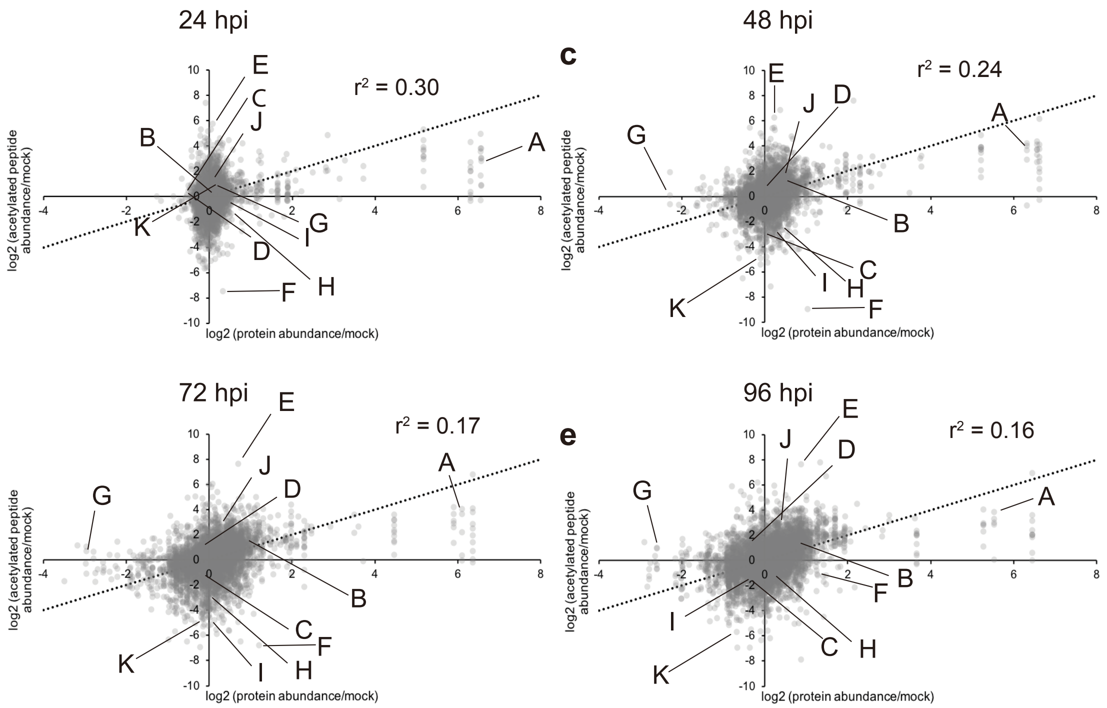

A: IFIT1 K192
B: ATP5B K133
C: LMNB1 K109
D: LMNB1 K134
E: PDHA1 K321
F: ME2 K24
G: RBX1 K105
H: RSF1 K1050
I: CREBBP K1583
J: ATP5B K519
K: HNRNPU K28

Supplementary Figure 4. Assessment of correlation between changes in acetylated peptide abundance and changes in protein abundance for host proteins. a Comparison of host acetylated peptides, the corresponding acetylated proteins, and the percentage of imputed values (used only in trend analyses in Figure 2b, Figure 3a, and Supplementary Figure 4b-e) across all three acetyl-IP biological replicates in all time points. These numbers represent the dataset of acetylated peptides that were filtered and used for quantitative analysis. **b-e** Dot plots of the log2 fold change of acetylated peptide abundance (normalized to mock) and corresponding protein abundance (normalized to mock) at **b** 24, **c** 48, **d** 72, and **e** 96 hpi. Each dot represents one

acetylated peptide, and a number of peptides of interest are marked. The dotted line indicates a slope equal to 1.

a

Cluster	# Proteins	Enriched GO Biological Processes	Representative Proteins	p-value	FDR
1	234	TCA cycle, response to virus, type I interferon signaling, gluconeogenesis	DLD, IFIT1, STAT1, GOT1	3.89E-14, 1.18E-05, 1.19E-05, 6.10E-05	5.11E-11, 1.74E-03, 1.74E-03, 4.45E-03
2	551	TCA cycle, glyoxylate metabolic process, tRNA aminoacylation for protein translation, canonical glycolysis, oxidative phosphorylation	PDHA, DLAT, MARS, ENO1, NDUFB6	2.22E-16, 8.40E-9, 5.11E-08, 1.03E-6, 1.82E-95	2.46E-13, 3.92E-06, 1.03E-05, 1.13E-04, 1.22E-03
3	641	translation, rRNA processing, cell-cell adhesion, TCA cycle, nucleosome assembly, glyoxylate metabolic process	RPL5, LDHA, ATP5O, SMARCA5, OGDH	1.11E-16, 1.11E-16, 1.11E-16, 4.28E-12, 1.19E-11, 5.57E-05	3.94E-14, 3.94E-14, 3.94E-14, 1.33E-09, 3.28E-09, 3.95E-03
4	833	translation, RNA processing, cell-cell adhesion, antigen processing and presentation, regulation of ubiquitin-protein ligase activity, NIK/NF-kappaB signaling, protein folding, viral process, canonical glycolysis, nucleosome assembly	RPL4, RUVBL1, MYO6, PSMD8, HSP90AB1, CREBBP, ENO1, KAT6B	1.11E-16, 1.11E-16, 1.11E-16, 1.11E-16, 1.11E-16, 5.77E-15, 2.93E-14, 2.49E-13, 9.94E-13, 6.69E-10, 1.27E-07, 5.85E-05	4.09E-14, 4.09E-14, 4.09E-14, 4.09E-14, 4.09E-14, 1.83E-12, 7.83E-12, 5.23E-11, 1.79E-10, 9.37E-08, 1.13E-05, 3.10E-03
5	662	translation, viral transcription, protein targeting to membrane, cell-cell adhesion, nucleosome assembly, mRNA splicing, histone H3 acetylation, protein stabilization	RPL4, FLNB, HIST2H2BF, HNRNPU, JADE1, HSP90AA1	1.11E-16, 1.11E-16, 1.11E-16, 1.11E-16, 2.67E-12, 1.59E-10, 8.56E-07, 5.24E-05	3.91E-14, 3.91E-14, 3.91E-14, 3.91E-14, 8.23E-10, 4.35E-08, 1.32E-04, 3.76E-03
6	329	cell-cell adhesion, translation, protein targeting to membrane, viral transcription, nucleosome assembly, rRNA processing, cell-junction assembly, regulation of mRNA stability	PFN1, EIF4A1, RPS9, H3F3A, ACTB, HNRNPD	2.19E-14, 2.13E-12, 2.82E-12, 7.14E-12, 3.00E-07, 4.77E-07, 6.45E-07, 2.52E-05	3.59E-11, 1.54E-09, 1.54E-09, 2.93E-09, 7.02E-05, 9.78E-05, 1.06E-04, 3.22E-03
7	230	beta-catenin-TCF complex assembly, nucleosome assembly	CREBBP, RSF1	7.34E-08, 6.81E-07	8.06E-05, 3.98E-04

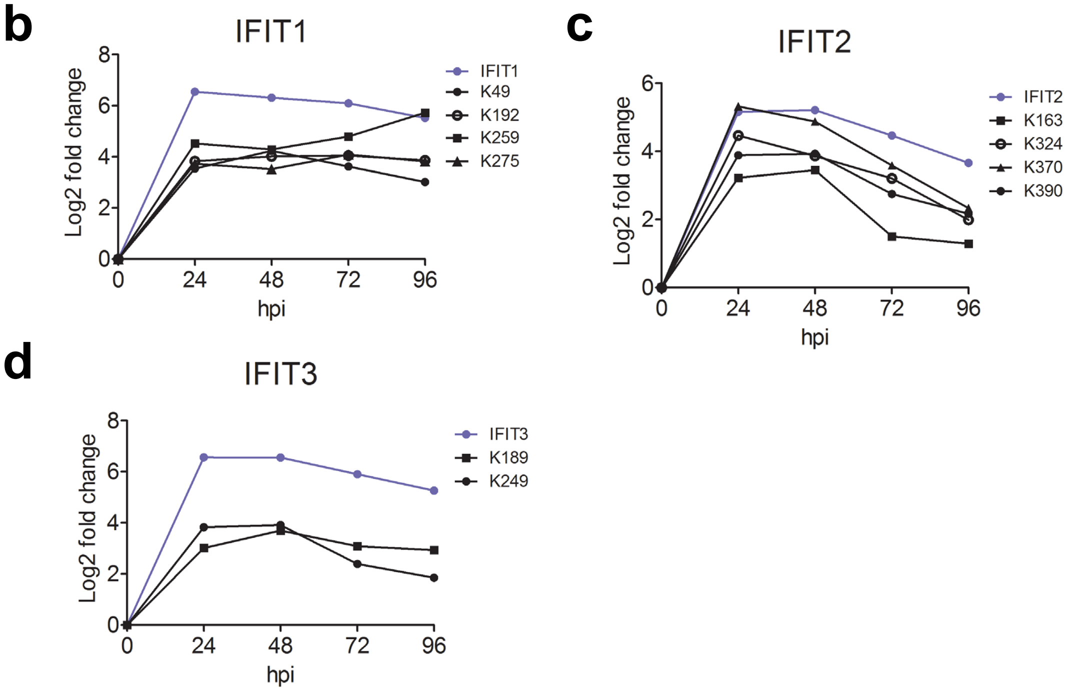

Supplementary Figure 5. Distinct acetylation abundance trends and different enriched GO Biological Processes. a Enriched GO Biological Processes with p-values ≤ E-05 were determined for each acetyl trend cluster through the Reactome plugin in Cytoscape. The p-value

and FDR for each term are shown in the same order as the terms are shown. **b-e** Comparison of log2 fold change of protein abundance to acetylated peptide abundance for IFIT proteins. IFIT protein abundance (normalized to mock) is shown in blue. Abundance of each acetylated peptide that was detected in both mock and infection time points is shown in black (normalized to mock). **b** IFIT1, **c** IFIT2, **d** IFIT3.

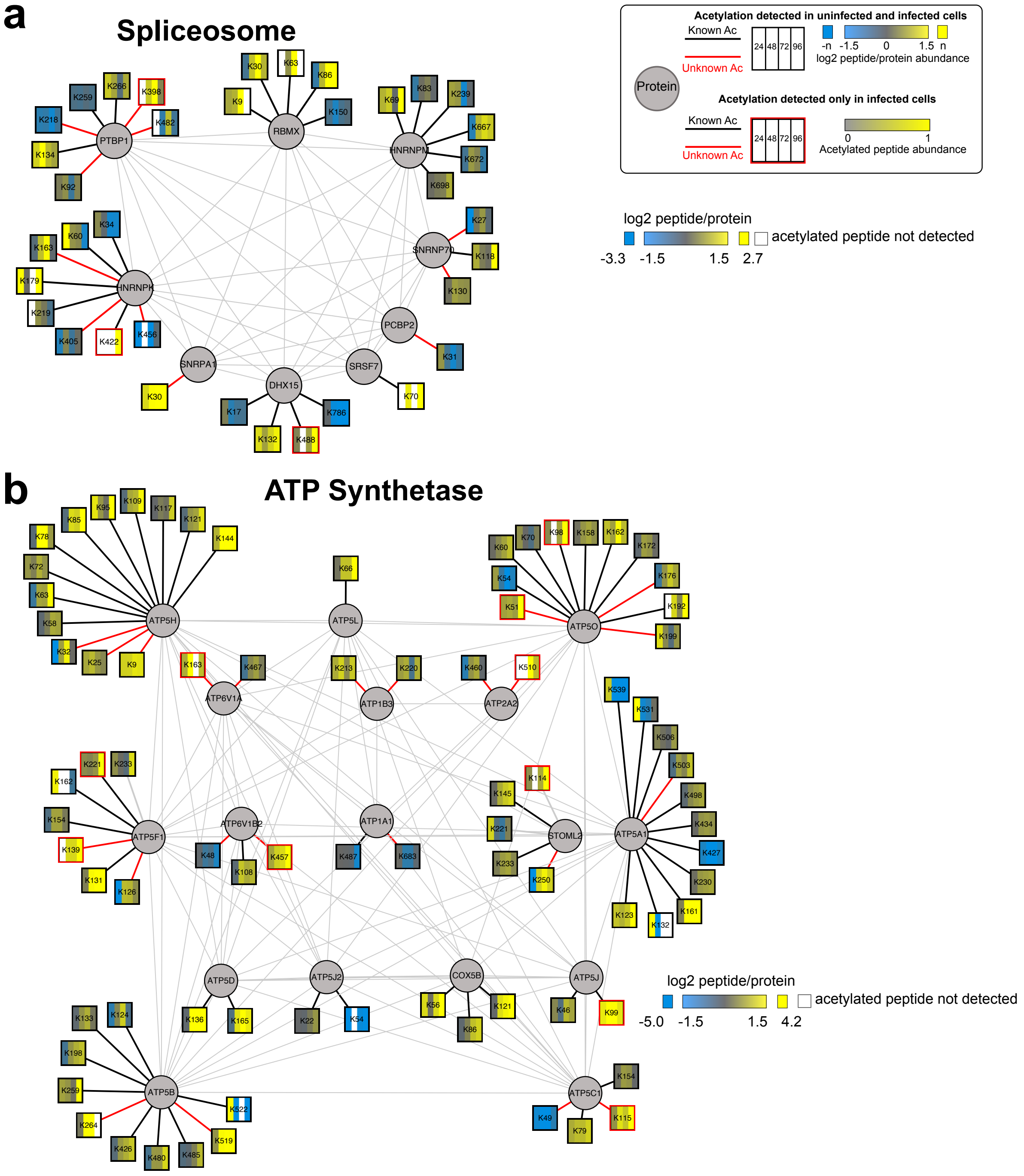

Supplementary Figure 6. Site-specific acetylation trends are observed on mRNA splicing and ATP synthetase proteins. a, b All detected acetylation sites on proteins from the indicated biological function representing different site-specific trends in acetylation during infection.

Log2 peptide/protein values are shown unless the acetylated peptide was not identified in mock, in which case the acetylated peptide abundances during infection are shown scaled 0 to 1. **a** Spliceosome **b** ATP synthetase complex. Circles, proteins; squares, acetylation sites; red lines, previously unknown acetylation sites; black lines, known acetylation sites; black boxes, detected in mock; red boxes, only detected during infection. If a peptide was not quantified in a given time point, the corresponding column within the acetyl box is white.

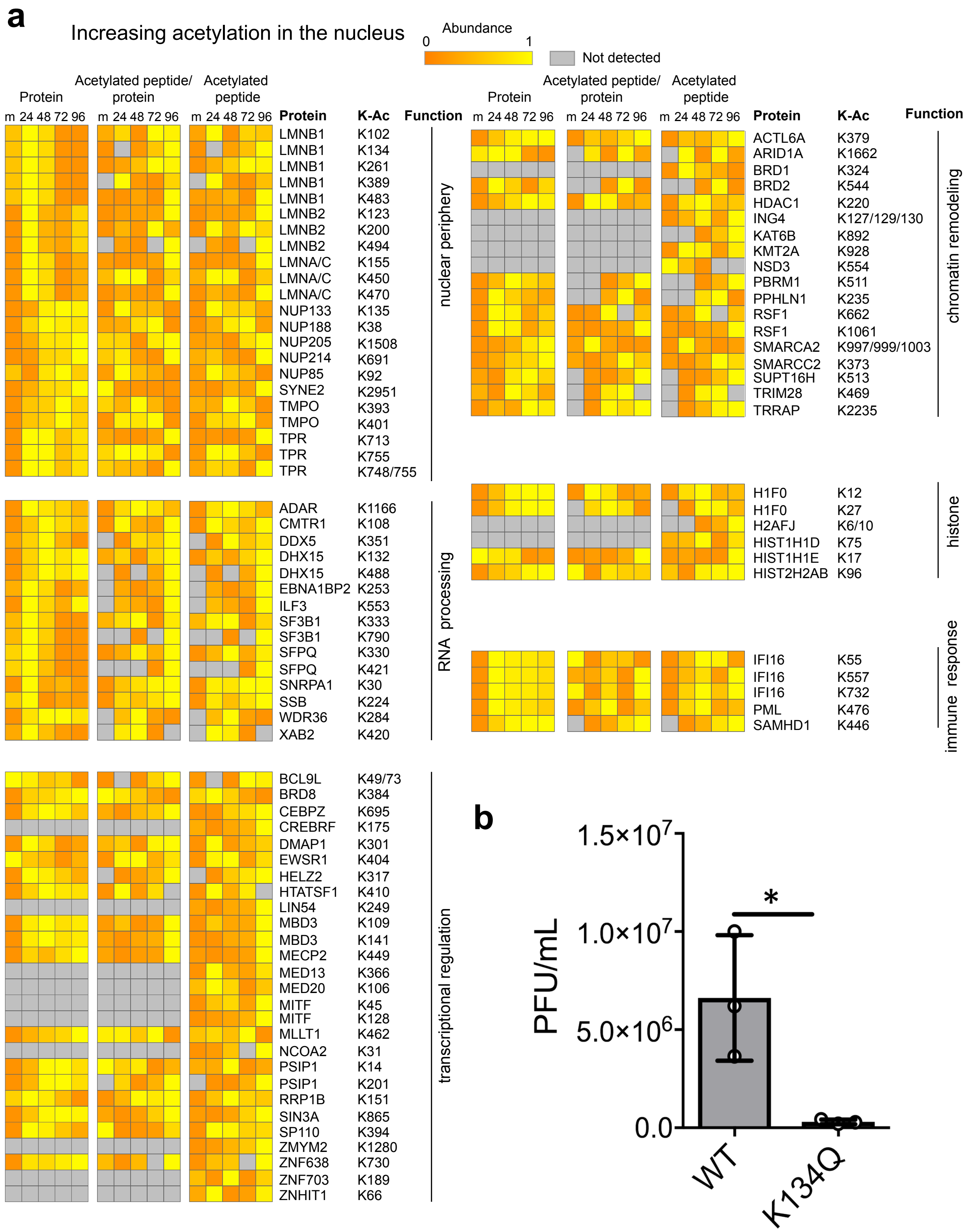
a
Increasing acetylation in the nucleus
Abundance
0
1
Not detected
Protein
Acetylated peptide/ protein
Acetylated peptide
m 24 48 72 96
Protein
K-Ac
Function
LMNB1 K102
LMNB1 K134
LMNB1 K261
LMNB1 K389
LMNB1 K483
LMNB2 K123
LMNB2 K200
LMNB2 K494
LMNA/C K155
LMNA/C K450
LMNA/C K470
NUP133 K135
NUP188 K38
NUP205 K1508
NUP214 K691
NUP85 K92
SYNE2 K2951
TMPO K393
TMPO K401
TPR K713
TPR K755
TPR K748/755
nuclear periphery
ADAR K1166
CMTR1 K108
DDX5 K351
DHX15 K132
DHX15 K488
EBNA1BP2 K253
ILF3 K553
SF3B1 K333
SF3B1 K790
SFPQ K330
SFPQ K421
SNRPA1 K30
SSB K224
WDR36 K284
XAB2 K420
RNA processing
BCL9L K49/73
BRD8 K384
CEBPZ K695
CREBRF K175
DMAP1 K301
EWSR1 K404
HELZ2 K317
HTATSF1 K410
LIN54 K249
MBD3 K109
MBD3 K141
MECP2 K449
MED13 K366
MED20 K106
MITF K45
MITF K128
MLLT1 K462
NCOA2 K31
PSIP1 K14
PSIP1 K201
RRP1B K151
SIN3A K865
SP110 K394
ZMYM2 K1280
ZNF638 K730
ZNF703 K189
ZNHIT1 K66
transcriptional regulation
ACTL6A K379
ARID1A K1662
BRD1 K324
BRD2 K544
HDAC1 K220
ING4 K127/129/130
KAT6B K892
KMT2A K928
NSD3 K554
PBRM1 K511
PPHLN1 K235
RSF1 K662
RSF1 K1061
SMARCA2 K997/999/1003
SMARCC2 K373
SUPT16H K513
TRIM28 K469
TRRAP K2235
chromatin remodeling
H1F0 K12
H1F0 K27
H2AFJ K6/10
HIST1H1D K75
HIST1H1E K17
HIST2H2AB K96
histone
IFI16 K55
IFI16 K557
IFI16 K732
PML K476
SAMHD1 K446
immune response
b
PFU/mL
1.5×10^7
1.0×10^7
5.0×10^6
0.0
*
WT
K134Q

Supplementary Figure 7. Acetylation is increased on a subset of nuclear proteins, including those at the nuclear periphery such as LMNB1. a Sites on nuclear proteins with increased levels of acetylation during infection clustered by function. Abundances are scaled from 0 to 1 (left, protein abundance; middle, acetylated peptide abundance normalized to protein abundance; right, acetylated peptide abundance). **b** $TCID_{50}$ assessment of the effect of LMNB1 K134Q mutant on infectious extracellular virus produced. Average of three replicates ± SD. One-sided Student's t-test was used. * $p < 0.05$.

```
                    1        10        20        30        40        50        60        70        80        90        100       110       120       130       140
human               MATATPVPP.RMGSRAGGPTTPLSPTRLSRLQEKEELRELNDRLAVYIDKVRSLETENSALQLQVTEREEVRGRELTGLKALYETELADARRALDDTARERAKLQIELGKCKAEHDQLLLNYAKKESDLNGAQIKLREYEAALNSKDAAL
chimpanzee          MATATPVPP.RMGSRAGGPTTPLSPTRLSRLQEKEELRELNDRLAVYIDKVRSLETENSALQLQVTEREEVRGRELTGLKALYETELADARRALDDTARERAKLQIELGKCKAEHDQLLLNYAKKESDLNGAQIKLREYEAALNSKDAAL
gorilla             MATATPVPP.RMGSRAGGPTTPLSPTRLSRLQEKEELRELNDRLAVYIDKVRSLETENSALQLQVTEREEVRGRELTGLKALYETELADARRALDDTARERAKLQIELGKCKAEHDQLLLNYAKKESDLNGAQIKLREYEAALNSKDAAL
sumatran_orangutan  MATATPVPP.RMGSRAGGPTTPLSPTRLSRLQEKEELRELNDRLAVYIDKVRSLETENSALQLQVTEREEVRGRELTGLKALYETELADARRALDDTARERAKLQIELGKCKAEHDQLLLNYAKKESDLNGAQIKLREYEAALNSKDAAL
olive_baboon        MATATPVPP.RTGSRAGGPTTPLSPTRLSRLQEKEELRELNDRLAVYIDKVRSLETENSALQLQVTEREEVRGRELTGLKALYETELADARRALDDTARERAKLQIELGKCKAEHDQLLLNYAKKESDLNGAQIKLREYEAALNSKDAAL
green_monkey        MATATPVPP.RTGSRAGGPTTPLSPTRLSRLQEKEELRELNDRLAVYIDKVRSLETENSALQLQVTEREEVRGRELTGLKALYETELADARRALDDTARERAKLQIELGKCKAEHDQLLLNYAKKESDLNGAQIKLREYEAALNSKDAAL
crab-eating_macaque MATATPVPP.RTGSRAGGPTTPLSPTRLSRLQEKEELRELNDRLAVYIDKVRSLETENSALQLQVTEREEVRGRELTGLKALYETELADARRALDDTARERAKLQIELGKCKAEHDQLLLNYAKKESDLNGAQIKLREYEAALNSKDAAL
rhesus_macaque      MATATPVPP.RTGSRAGGPTTPLSPTRLSRLQEKEELRELNDRLAVYIDKVRSLETENSALQLQVTEREEVRGRELTGLKALYETELADARRALDDTARERAKLQIELGKCKAEHDQLLLNYAKKESDLNGAQIKLREYEAALNSKDAAL
mouse               MATATPVQQQRAGSRASAPATPLSPTRLSRLQEKEELRELNDRLAVYIDKVRSLETENSALQLQVTEREEVRGRELTGLKALYETELADARRALDDTARERAKLQIELGKFKAEHDQLLLNYAKKESDLSGAQIKLREYEAALNSKDAAL
rat                 MATATPVQQ.RAGSRASAPATPFSPTRLSRLQEKEELRELNDRLAVYIDKVRSLETENSALQLQVTEREEVRGRELTGLKALYETELADARRALDDTARERAKLQIELGKFKAEHDQLLLNYAKKESDLSGAQIKLREYEAALNSKDAAL
bovine              MATATPVPP.RSGSRAGGPATPLSPTRLSRLQEKEELRELNDRLAVYIDKVRSLETENSALQLQVTEREEVRGRELTGLKALYETELADARRALDDTARERAKLQIELGKFKAEHDQLLLNYAKKESDLNGAQIKLREYEAALNSKDAAL
Western_clawed_frog MATATPSGP.RSSGRRSSMNTPLSPTRISRLQEKVDLQELNDRLAVYIDKVRSLESENSLLHVQVTEREEVRSREVSGIKELYETELADARRSLDDTARERARLQLELSKISVEHEDLRASFSKKESELESAQARFRETEALLNSKNAAL
African_clawed_frog MATATPSGP.RSSGRRSSMSTPLSPTRITRLQEKVDLQELNDRLALYIDTVRSLESENSLLHVQVTEREEVRSREVSGIKELYETELADARRSLDDTAREKARLQLELSKVSVEHQDLQASFSKRESELESTQARFRETEALLNSKNAAL
zebrafish           MASAACTPAGQQSLSRGTSSTPLSPARISRLQEKEELQQLNDRLAAYIDTVRSLESENSVLQIQIREREDVRNRELTGLRTLYETELADARKALDDTARERARLQIELGKIKSEQEQLQHTYAKKDADLTSSQNRLKDVEALLNAKEAAL
turquoise_killifish MATTTATPTSHKGGTCRG.GTPLSPTRITRLQEKQQLRDLNDRLAVYIDKVRSLESENEVLHLQISEKEEVRSRELSGVRSLYETELADARRSLDDSSKERAWLQIELGKIRSEHELLQQNYARKDSEAAGAQARLRDLEAQLNSKDALL
bluefin_notho       MATTTATPASHKGGTCRG.GTPLSPTRITRLQEKQQLRDLNDRLAVYIDKVRSLESENEVLHLQISEKEEVRSRELSGVRSLYETELADARRSLDDSSKERAWLQIELGKIRSEHELLQQNYARKDSEAAGAQARLRDLEAQLNSKDALL

                    150       160       170       180       190       200       210       220       230       240       250       260       270       280       290
human               ATALGDKKSLEGDLEDLKDQIAQLEASLAAAKKQLADETLLKVDLENRCQSLTEDLEFRKSMYEEEINETRRKHETRLVEVDSGRQIEYEYKLAQALHEMREQHDAQVRLYKEELEQTYHAKLENARLSSEMNTSTVNSAREELMESRMR
chimpanzee          ATALGDKKSLEGDLEDLRDQIAQLEASLAAAKKQLADETLLKVDLENRCQSLTEDLEFRKNMYEEEINETRRKHETRLVEVDSGRQIEYEYKLAQALHEMREQHDAQVRLYKEELEQTYHAKLENARLSSEMNTSTVNSAREELMESRMR
gorilla             ATALGDKKSLEGDLEDLKDQIAQLEASLAAAKKQLADETLLKVDLENRCQSLTEDLEFRKNMYEEEINETRRKHETRLVEVDSGRQIEYEYKLAQALHEMREQHDAQVRLYKEELEQTYHAKLENARLSSEMNTSTVNSAREELMESRMR
sumatran_orangutan  ATALGDKKSLEGDLEDLKDQIAQLEASLAAAKKQLADETLLKVDLENRCQSLTEDLEFRKNMYEEEINETRRKHETRLVEVDSGRQIEYEYKLAQALHEMREQHDAQVRLYKEELEQTYHAKLENARLSSEMNTSTVNSAREELMESRMR
olive_baboon        ATALGDKKSLEGDLEDLKDQIAQLEASLAAAKKQLADETLLKVDLENRCQSLTEDLEFRKNMYEEEINETRRKHETRLVEVDSGRQIEYEYKLAQALHEMREQHDAQVRLYKEELEQTYHAKLENARLSSEMNTSTVNSAREELMESRMR
green_monkey        ATALGDKKSLEGDLEDLKDQIAQLEASLAAAKKQLADETLLKVDLENRCQSLTEDLEFRKNMYEEEINETRRKHETRLVEVDSGRQIEYEYKLAQALHEMREQHDAQVRLYKEELEQTYHAKLENARLSSEMNTSTVNSAREELMESRMR
crab-eating_macaque ATALGDKKSLEGDLEDLKDQIAQLEASLAAAKKQLADETLLKVDLENRCQSLTEDLEFRKNMYEEEINETRRKHETRLVEVDSGRQIEYEYKLAQALHEMREQHDAQVRLYKEELEQTYHAKLENARLSSEMNTSTVNSAREELMESRMR
rhesus_macaque      ATALGDKKSLEGDLEDLKDQIAQLEASLAAAKKQLADETLLKVDLENRCQSLTEDLEFRKNMYEEEINETRRKHETRLVEVDSGRQIEYEYKLAQALHEMREQHDAQVRLYKEELEQTYHAKLENARLSSEMNTSTVNSAREELMESRMR
mouse               ATALGDKKSLEGDLEDLKDQIAQLEASLSAAKKQLADETLLKVDLENRCQSLTEDLEFRKNMYEEEINETRRKHETRLVEVDSGRQIEYEYKLAQALHEMREQHDAQVRLYKEELEQTYHAKLENARLSSEMNTSTVNSAREELMESRMR
rat                 ATALGDKKSLEGDLEDLKDQIAQLEASLSAAKKQLADETLLKVDLENRCQSLTEDLEFRKNMYEEEINETRRKHETRLVEVDSGRQIEYEYKLAQALHEMREQHDAQVRLYKEELEQTYHAKLENARLSSEMNTSTVNSARGGMMESRMR
bovine              ATALGDKKSLESDLEDLKDQIAQLEASLAAAKKQLADETLLKVDLENRCQSLTEDLEFRKNMYEEEINETRRKHETRLVEVDSGRQIEYEYKLAQALHEMREQHDAQVKLYKEELEQTYHAKLENARLSSEMNTSTVNSAREELMESRMR
Western_clawed_frog ATTQSENKSLQGEVEDLKAEIGQLESALALAKKQLEEEILIKVDLENRCQSLIEELDFRKNVYEEEIKETRRRHETRLVEVDSGKQIEYEHKLSQALADMREQHDSQVTLYKEELEHTYQSKLENARLSSEMNSSAVNSTREELMESRYR
African_clawed_frog ATAQSENKSLQGEVEDLKAEIGQLGSALALAKKQLEEEILMKVDLENRCQSLIEELNFRKNIYEEEIKETSRRHETRLVEVDSGRQVDYEYKLSQALSEMREQQESQIGLYKEELEQTYQSKLENARLASEMNSSAVNSTREELMESRIR
zebrafish           NTAVSERKTLENTLSDLQIRVQELEGGLISAKKQLSDETLLRVDLENRCQSLMEELEFRKNMFEEEIKDTRRRHETRLVEVDSGRQMEYEFKLAQALTDMRQQHDEQVKLYKEEMEQTYVSKLENIRLSSEMNSSSASMAREELRESTLR
turquoise_killifish ATALSEKRALEASLAELQEQLQELDIGLAQAKKQLADEMLMRVDLENRCQSMTEEMEFQKNIHAEEVKESRQRYETRLVEVDSGRKEEYEYKLTQALADMRAQNEEQILIYKDSLESTYKAKLEDLHRLSEMNGSSANMAREELRESALR
bluefin_notho       ATALSEKRALEASLAELQEQLQELDIGLAQAKKQLADEMLMRVDLENRCQSMTEEMEFQKNIHAEEVKEARQRYETRLVEVDSGRKEEYEYKLTQALADMRAQNEEQILIYKDSLESTYKAKLEDLHRLSEMNGSSANMAREELRESALR

                    300       310       320       330       340       350       360       370       380       390       400       410       420       430       440
human               IESLSSQLSNLQKESRACLERIQELEDLLAKEKDNSRRMLTDKEREMAEIRDQMQQQLNDYEQLLDVKLALDMEISAYRKLLEGEEERLKLSPSPSSRVTVSRASSS.RSVRTTRGKRKRVDVEESEASSSVSISHSASATGNVCIEEID
chimpanzee          IESLSSQLSNLQKESRACLERIQELEDLLAKEKDNSRRMLTDKEREMAEIRDQMQQQLNDYEQLLDVKLALDMEISAYRKLLEGEEERLKLSPSPSSRVTVSRASSS.RSVRTTRGKRKRVDVEESEASSSVSISHSASATGNVCIEEID
gorilla             IESLSSQLSNLQKESRACLERIQELEDLLAKEKDNSRRMLTDKEREMAEIRDQMQQQLNDYEQLLDVKLALDMEISAYRKLLEGEEERLKLSPSPSSRVTVSRASSS.RSVRTTRGKRKRVDVEESEASSSVSISHSASATGNVCIEEID
sumatran_orangutan  IESLSSQLSNLQKESRACLERIQELEDLLAKEKDNSRRMLTDKEREMAEIRDQMQQQLNDYEQLLDVKLALDMEISAYRKLLEGEEERLKLSPSPSSRVTVSRASSS.RSVRTTRGKRKRVDVEESEASSSVSISHSASATGNVCIEEID
olive_baboon        IESLSSQLSNLQKESRACLERIQELEDMLAKEKDNSRRMLTDKEREMAEIRDQMQQQLNDYEQLLDVKLALDMEISAYRKLLEGEEERLKLSPSPSSRVTVSRASSS.RSVRTTRGKRKRVDVEESEASSSVSISHSASATGNVCIEEID
green_monkey        IESLSSQLSNLQKESRACLERIQELEDMLAKEKDNSRRMLTDKEREMAEIRDQMQQQLNDYEQLLDVKLALDMEISAYRKLLEGEEERLKLSPSPSSRVTVSRASSS.RSVRTTRGKRKRVDVEESEASSSVSISHSASATGNVCIEEID
crab-eating_macaque IESLSSQLSNLQKESRACLERIQELEDMLAKEKDNSRRMLTDKEREMAEIRDQMQQQLNDYEQLLDVKLALDMEISAYRKLLEGEEERLKLSPSPSSRVTVSRASSS.RSVRTTRGKRKRVDVEESEASSSVSISHSASATGNVCIEEID
rhesus_macaque      IESLSSQLSNLQKESRACLERIQELEDMLAKEKDNSRRMLTDKEREMAEIRDQMQQQLNDYEQLLDVKLALDMEISAYRKLLEGEEERLKLSPSPSSRVTVSRASSS.RSVRTTRGKRKRVDVEESEASSSVSISHSASATGNVCIEEID
mouse               IESLSSQLSNLQKESRACLERIQELEDMLAKERDNSRRMLSDREREMAEIRDQMQQQLSDYEQLLDVKLALDMEISAYRKLLEGEEERLKLSPSPSSRVTVSRASSS.RSVRTTRGKRKRVDVEESEASSSVSISHSASATGNVCIEEID
rat                 IESLSSQLSNLQKDSRACLERIQELEDMLAKERDNSRRMLSDKEREMAEIRDQMQQQLNDYEQLLDVKLALDMEISAYRKLLEGEEERLKLSPSPSSRVTVSRASSS.RSVRTTRGKRKRVDVEESEASSSVSISHSASATGNVCIEEID
bovine              IESLSSQLSNLQKESRACLERIQELEDLLAKERDNSRRMLSDKEREMAEIRDQMQQQLNDYEQLLDVKLALDMEISAYRKLLEGEEERLKLSPSPSSRVTVSRASSS.RSVRTTRGKRKRVDVEESEASSSVSISHSASATGNVCIEEID
Western_clawed_frog IDSLTSQLSELQKESRAWHDRMQELEDMLAKEKDNSRKMLAEREREMADIRDQMQQQLNDYEQLLDVKLALDMEISAYRKLLEGEEERLKLSPSPPSRVTVSRASSS.RSVRTAKGKRKRIDVEESEASSSVSISHSASATGDVSIEEVD
African_clawed_frog IDSLTSQLSELQKESRAWHDRMQELEDMLAKEKDNSRKMLAEREREMADIRDQMQQQLNDYEQLLDVKLALDMEISAYRKLLEGEEERLKLSPSP.SRVTVSRASSS.RAVRTTKGKRKRIDVEESEASSSVSIDHSAAATGDVSIEEVD
zebrafish           VESLAGQLANLQKEARAWQDRINELEGALSHEKDLSRRLLAEKEREIAEIRAKMQQQLDEYEQLLDVKLALDMEINAYRKLLEGEEERLKLSPSPSSRVTVSRASSS.RSVRTTRGKRKRVDVEESEASSSVSIAHSASATGNVSIDELD
turquoise_killifish IESLSAQLAGLQKETRGWRDRILELEVALTQEKDRSRKLLSDKDREVSEIRAKMQQQLNEYEQLLDVKLALDMEINAYRKLLEGEEERLKLSPSPSSRVTVSRASSSSRSVRTARGKRKRIDVEEQEASSSVSIAHSASATGPIVVDEID
bluefin_notho       IESLSAQLAGLQKETRGWRDRILELEAALTQEKDRSRKLLTDKDREVSEIRAKMQQQLNEYEQLLDVKLALDMEINAYRKLLEGEEERLKLSPSPSSRVTVSRASSSSRSVRTARGKRKRIDVEEQEASSSVSIAHSASATGPIVVDEID

                    450       460       470       480       490       500       510       520       530       540       550       560       570       580
human               VDGKFIRLKNTSEQDQPMGGWEMIRKIGDTSVSYKYTSRYVLKAGQTVTIWAANAGVTASPPTDLIWKNQNSWGTGEDVKVILKNSQGEEVAQRSTVFKTTIPEEEEEEEEAA.GVVVEEELFHQQGTP.RASNRSCAIM
chimpanzee          VDGKFIRLKNTSEQDQPMGGWEMIRKIGDTSVSYKYTSRYVLKAGQTVTIWAANAGVTASPPTDLIWKNQNSWGTGEDVKVILKNSQGEEVAQRSTVFKTTIPEEEEEEEEAA.GVVVEEELFHQQGTP.RASNRSCAIM
gorilla             VDGKFIRLKNTSEQDQPMGGWEMIRKIGDTSVSYKYTSRYVLKAGQTVTIWAANAGVTASPPTDLIWKNQNSWGTGEDVKVILKNSQGEEVAQRSTVFKTTIPEEEEEEEEAA.GVVVEEELFHQQGTP.RASNRSCAIM
sumatran_orangutan  VDGKFIRLKNTSEQDQPMGGWEMIRKIGDTSVSYKYTSRYVLKAGQTVTIWAANAGVTASPPTDLIWKNQNSWGTGEDVKVILKNSQGEEVAQRSTVFKTTIPEEEEEEEEAA.GVVVEEELFHQQGTP.RTSNRSCAIM
olive_baboon        VDGKFIRLKNTSEQDQPMGGWEMIRKIGDTSVSYKYTSRYVLKAGQTVTIWAANAGVTASPPTDLIWKNQNSWGTGEDVKVILKNSQGEEVAQRSTVFKTTIPEEEEEEEEAA.GVVVEEELFHQQGTP.RTSNRSCAIM
green_monkey        VDGKFIRLKNTSEQDQPMGGWEMIRKIGDTSVSYKYTSRYVLKAGQTVTIWAANAGVTASPPTDLIWKNQNSWGTGEDVKVILKNSQGEEVAQRSTVFKTTIPEEEEEEEEAA.GVVVEEELFHQQGTP.RTSNRSCAIM
crab-eating_macaque VDGKFIRLKNTSEQDQPMGGWEMIRKIGDTSVSYKYTSRYVLKAGQTVTIWAANAGVTASPPTDLIWKNQNSWGTGEDVKVILKNSQGEEVAQRSTVFKTTIPEEEEEEEEAA.GVVVEEELFHQQGTP.RTSNRSCAIM
rhesus_macaque      VDGKFIRLKNTSEQDQPMGGWEMIRKIGDTSVSYKYTSRYVLKAGQTVTIWAANAGVTASPPTDLIWKNQNSWGTGEDVKVILKNSQGEEVAQRSTVFKTTIPEEEEEEEEAA.GVVVEEELFHQQGYS.R.........
mouse               VDGKFIRLKNTSEQDQPMGGWEMIRKIGDTSVSYKYTSRYVLKAGQTVTVWAANAGVTASPPTDLIWKNQNSWGTGEDVKVILKNSQGEEVAQRSTVFKTTIPEEEEEEEEEEPIGVAVEEERFHQQGAP.RASNKSCAIM
rat                 VDGKFIRLKNTSEQDQPMGGWEMIRKIGDTSVSYKYTSRYVLKAGQTVTVWAANAGVTASPPTDLIWKNQNSWGTGEDVKVVLKNSQGEEVAQRSTVFKTTIPEEEEEEEEEEPIGVPLEEERFHQQGTP.RASNKSCAIM
bovine              VDGKFIRLKNTSEQDQPMGGWEMIRKIGDTSVSYKYTSRYVLKAGQTVTVSGPSIGVSASAPPDSMWKVRNEWG.GMLVGVLLRGSCAEEVAQRSTVFKTTIPEEEEEEEEAA.EVAVEEELFHQQGAP.RGSNRSCAIM
Western_clawed_frog VDGKYIRLKNNSEQDHPLGGWELTRTIGEASVNFKFTSRYVLKAGQTVTIWAADAGVTASPPSDLIWKNQNSWATGEDVKVILKNSQGEDVAQRTTVYTTTIPEEELEEEEEMLEEITKESHYSQQQTPQKSGNRNCAIM
African_clawed_frog VDGKYIRLKNNSEKDHPLGGWELTRTIGEASVNFKFTSRYVLKAEQTVTIWAADAGVKASPPSDLIWKNQNSWGTGEDVKATLKNSQGEEVAQRTTIYTTNIPEEEFEEGEEIFEETAKEFHYPQQ....KSGNKNCAIM
zebrafish           VDGKFIRLHNNSEQDQPMAGFQLTRTIGEVTATYKFTAKYNLKAGQKVTIWASNAGISSNPPADLIWKNQPSWGTGENIKVALLSPAGEKVAVRSTVFKTAVEDEDDDEEEEKEAEVIEERFYHQQGDP.RAAKRGCLIM
turquoise_killifish TDGKFICLHNTGDEDQALVGYEMVKTIGNTSATYKFTPKYVLKAGQKVMIWASDAGVSSKPPMDLVWKSQNSWGSGEDVSVVLMNPQGEEVAKRTTTYKTSIDQVEEYDEVE....VIEEELFQQQGGS.GSPKRGCAIM
bluefin_notho       TDGKFICLHNTGDEDQALVGYEMVKTIGNTSATYKFTPKYVLKAGQKVMIWASDAGVSSKPPMDLVWKSQNSWGSGEDVSVVLMNPQGEEVAKRTTTYKTSIDQVEEYDEVE....VIEEELFQQQGGS.GSSKRGCAIM
```

Supplementary Figure 8. Sequence alignment of identified LMNB1 acetylated lysines and regulatory phosphorylated serines from different vertebrate species. Protein amino acid sequences from 16 species were retrieved from Uniprot, aligned by MultAlin, and colored by ESPript3. The conserved acetylated lysine sites detected in this study are highlighted in yellow and conserved phosphorylated serine sites of known function are highlighted in cyan.

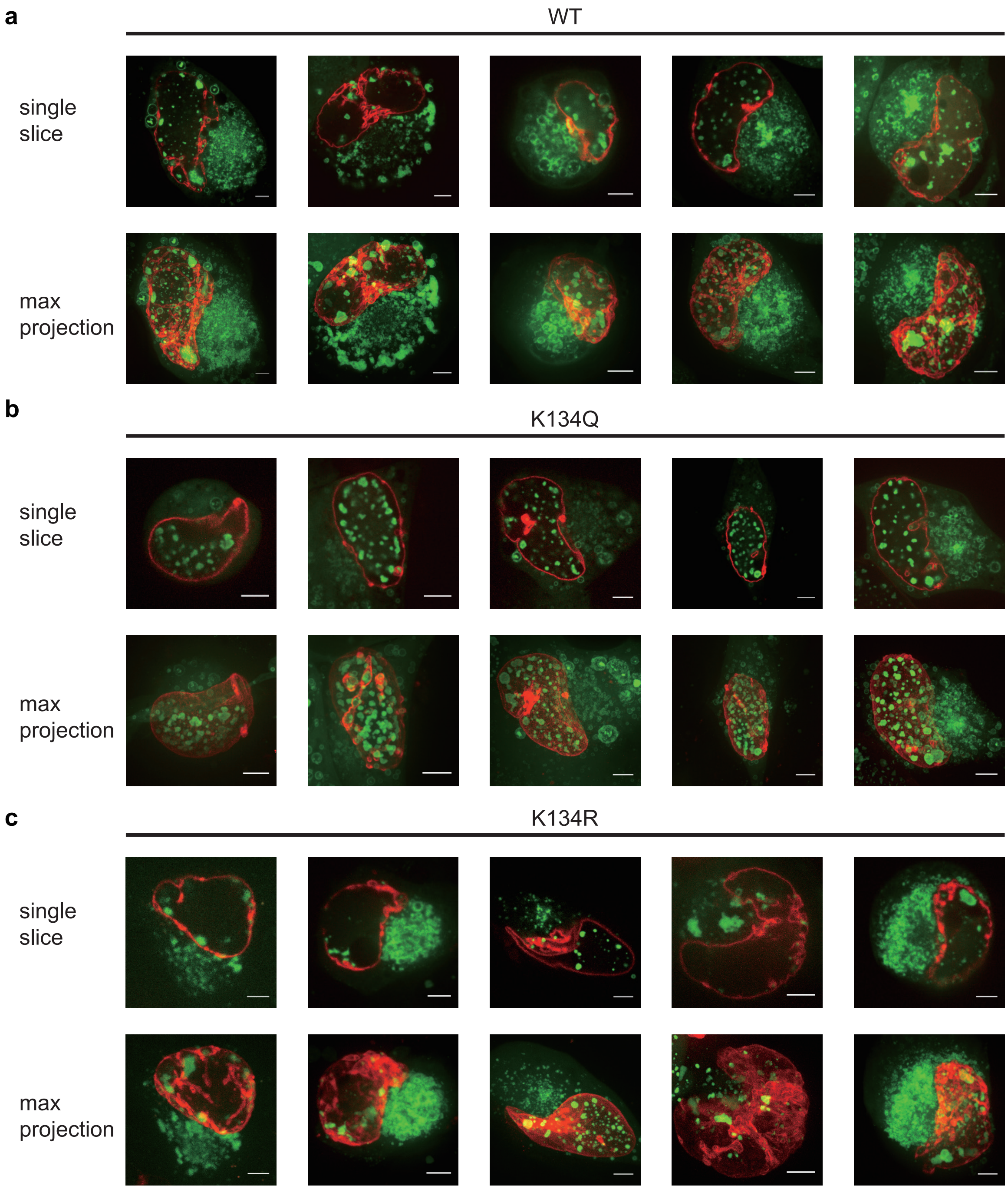

Supplementary Figure 9. Acetylation of LMNB1 impedes nuclear capsid egress. Additional examples of live cell confocal fluorescence microscopy images of cells transfected with **a** mCherry-LMNB1 WT, **b** K134Q, or **c** K134R (red) and infected with HCMV AD169 UL32-

GFP (green) at 120 hpi. Five representative images for each construct are shown as a single slice through the center of the Z-stack (upper row) and as a maximum projection of the Z-stack (bottom row). The rightmost images for each construct are the single slice and maximum projection that correspond to the images shown in Figure 5a; scale bar = 5 µm.

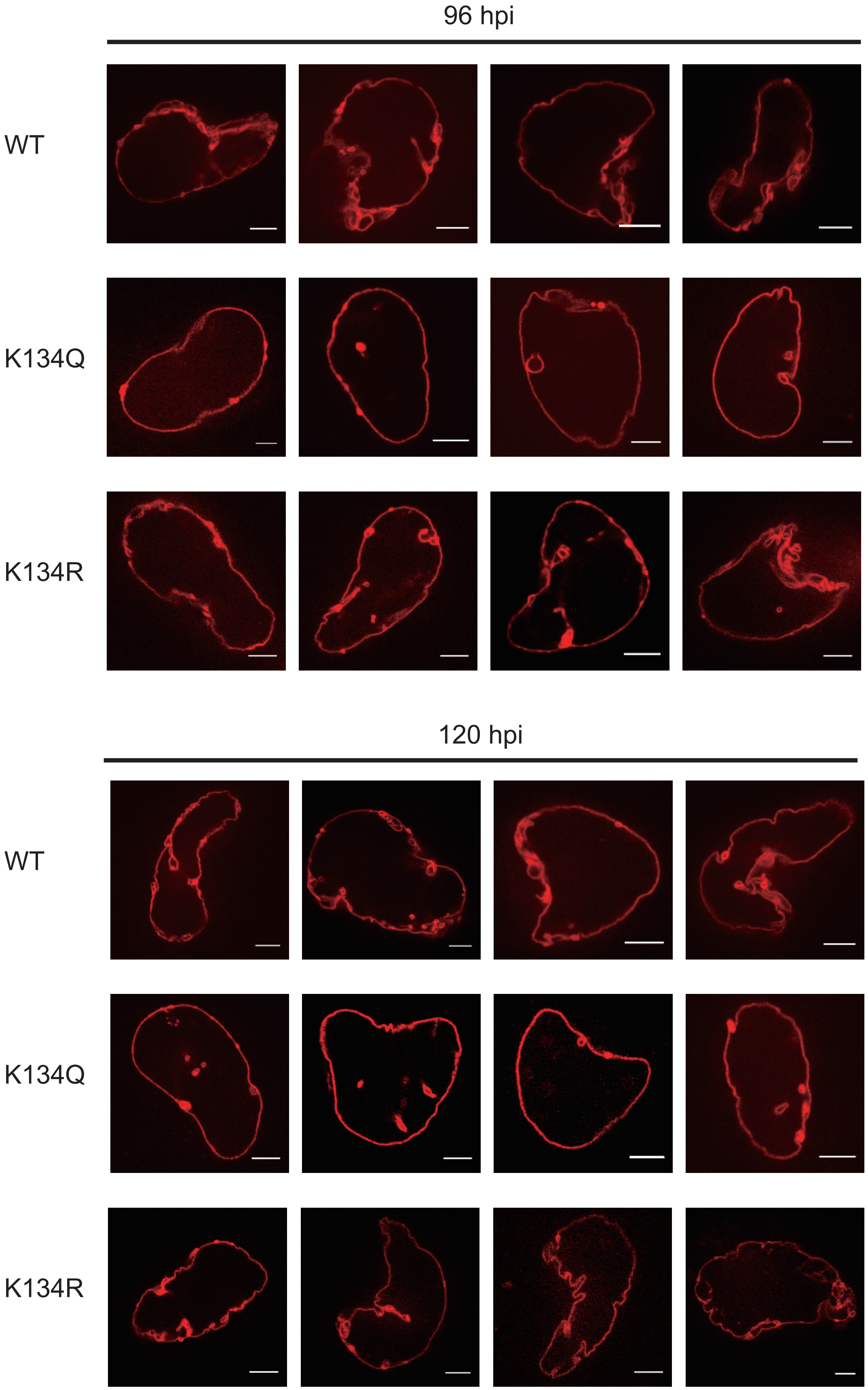
96 hpi
WT
K134Q
K134R
120 hpi
WT
K134Q
K134R

Supplementary Figure 10. Acetylation at K134 on LMNB1 decreases nuclear periphery disruption. Cells transfected with mCherry-LMNB1 WT, K134Q, or K134R (red) and infected with HCMV were imaged by live cell confocal fluorescence microscopy at 96 and 120 hpi. Four representative images for each LMNB1 construct per time point are shown as a single slice through the center of the Z-stack; scale bar = 5 μm.

a

Replicate	24 hpi rep1	24 hpi rep2	24 hpi rep3	48 hpi rep1	48 hpi rep2	48 hpi rep3	72 hpi rep1	72 hpi rep2	72 hpi rep3	96 hpi rep1	96 hpi rep2	96 hpi rep3
# acetylated peptides	17	10	19	15	13	27	18	13	29	24	16	31
# acetylated proteins	12	8	13	11	9	17	13	9	19	14	11	19

b

Replicate	24 hpi rep1	24 hpi rep2	24 hpi rep3	48 hpi rep1	48 hpi rep2	48 hpi rep3	72 hpi rep1	72 hpi rep2	72 hpi rep3	96 hpi rep1	96 hpi rep2	96 hpi rep3
# proteins	75	73	77	79	79	88	83	85	90	89	90	91
# peptides detected	376	380	482	527	528	791	680	766	944	787	917	1007
# peptides quantified	349	354	406	504	504	584	660	739	767	761	885	912

c

Capsid

Protein	Domain region (aa)	Domain region function	Protein length (aa)	Temporal expression	Acetylated lysine(s)
UL46			290	L	25, 270
UL85			306	L	15, 23, 166
UL86	1-59<br>60-189, 234-290, 363-397, 1032-1106<br>190-233<br>291-362<br>398-480, 1322-1370<br>481-1031<br>1107-1321	N-lasso<br>Johnson fold<br>helix-hairpin<br>dimerization<br>channel<br>upper region<br>buttress	1370	L	24, 85, 118, 165, 220, 377, 413, 415, 439, 513, 1257, 1280, 1336

Envelope

Protein	Domain region (aa)	Domain region function	Protein length (aa)	Temporal expression	Acetylated lysine(s)
gB	1-31<br>92-111<br>152-158<br>237-244<br>411-447<br>696-748<br>894-897	signal peptide<br>disintegrin-like domain<br>involved in fusion and/or binding to host membrane<br>involved in fusion and/or binding to host membrane<br>required to assemble an antigenic region<br>hydrophobic membrane proximal region<br>internalization motif	906	DE	230, 370, 633, 669, 699
gM (UL100)	1-13<br>14-34<br>35-79<br>80-100<br>101-126<br>127-147<br>148-151<br>152-172<br>173-200<br>201-221<br>222-239<br>240-260<br>261-264<br>265-285<br>286-298<br>299-319<br>320-372<br>300-372<br>1-118	intravirion<br>helical<br>virion surface<br>helical<br>intravirion<br>helical<br>virion surface<br>helical<br>intravirion<br>helical<br>virion surface<br>helical<br>intravirion<br>helical<br>virion surface<br>helical<br>intravirion<br>interacts with FIP4<br>region that binds gN	372	L	8, 108, 331
gL	1-30	signal peptide	278	L	248
gH (UL75)	24-270<br>218-281<br>721-741<br>742-743	virion surface<br>interacts with gL<br>helical<br>intravirion	743	L	131, 327, 405
gO (UL74)			466	L	188
IRL10	80-100	helical	171	DE	35
IRL11	24-294<br>295-317<br>318-345	virion surface<br>helical<br>intravirion	345	DE	175

Tegument

Protein	Domain region (aa)	Domain region function	Protein length (aa)	Temporal expression	Acetylated lysine(s)
UL25			656	L	609
UL26	1-34<br>184-222	important for viral growth<br>essential for replication	222	DE	203
UL32	1-275<br>982-1048	capsid (SCP) binding region<br>C terminal interacts with UL96	1048	DE	237, 373, 394, 805, 903, 1000
UL35			640	DE	118
UL43			423	L	135
UL44	1-309<br>1-290<br>162-174<br>425-431<br>40-433<br>291-433<br>129-140<br>1-290<br>1-200, 313-433	needed for function as a processivity factor<br>self-dimerization<br>highly flexible loop, involved in DNA binding<br>NLS<br>interacts with UL112/113<br>interacts with UL114<br>connector loop, interacts with UL54<br>interacts with UL84<br>interacts with Ubc9	433	DE	101, 288, 338, 376
UL45			906	DE	523, 614
UL47	474-983	interaction with UL48	983	L	281
UL48	1-238<br>322-754	deubiquitination activity<br>interacts with UL47	2241	DE	439, 465, 481, 595, 649, 809, 1001, 1003, 1019, 1175, 1261, 1288, 1478
UL50	52-60, 125-139<br>1-169	heterodimerization with UL53<br>conserved	397	DE	20, 132
UL54	379-421<br>492-588<br>696-742<br>771-790<br>805-845<br>905-919<br>962-970<br>978-988<br>1153-1159<br>1222-1227<br>1220-1242<br>1-1212	conserved with other DNA-dependent DNA polymerases (labeled in aa order): (IV, δ-region C, II, VI, III, I, VII, V); I, II, III: binding of deoxynucleoside triphosphates, chelating the Mg2+ ion, and interacting with primer and template; IV and δ-region C: 3' to 5' exonuclease function<br>NLSA<br>NLSB<br>interaction with UL44<br>interaction with UL114	1242	DE	500
UL69	17-30<br>35-46, 122-139<br>387-501<br>596-624	UAP56 interaction<br>RNA binding domain<br>zinc finger CHC2-type<br>NES	744	L	275, 580
UL82			559	L	91, 376
UL83	1-386<br>387-470<br>471-561	pyrin association domain<br>linker<br>conserved C terminal domain and NLS	561	L	154, 176, 191, 215, 283, 378, 509, 538, 557
UL84	1-68<br>101-200<br>161-170<br>229-238<br>360-367	association with pUL44 and viral DNA replication<br>interaction with CK2, essential for DNA replication<br>nuclear localization signal<br>nuclear export signal 1<br>nuclear export signal 2	587	DE	94, 339, 436
UL94			345	DE	306, 316, 324
UL96	1-101<br>122-127	interaction with UL32<br>critical for virus maturation	127	DE	10
UL97	48-110<br>292-707<br>337-345<br>352-360<br>373-381<br>433-450	NLS<br>important for phosphorylation activity<br>ATP binding/ Domain I (sim to PK)<br>Domain II (sim to PK)<br>Domain III (sim to PK)<br>Domain VIa (sim to PK)	707	DE	586
UL122	200-208	non-covalent SUMO1 binding region	580	IE	21, 161, 337, 421
US22			576	DE	197, 305, 356

Supplementary Figure 11. Viral acetylome. a Comparison of the number of viral acetylated peptides and the corresponding acetylated viral proteins across all three acetyl-IP biological replicates in all infection time points. **b** Comparison of the number of viral proteins and the number of corresponding detected and quantified unmodified peptides across all three biological

replicates in all infection time points from the whole proteome dataset. **c** Functional domains, temporal expression, and acetylated lysine sites detected in this study on HCMV proteins known to be part of the capsid, envelope, and tegument. All domain information is annotated from Uniprot and primary literature.
