# Supplementary material for: Orchestration of protein acetylation as a toggle for cellular defense and virus replication
Source: Nat Commun. 2018 Nov 23;9:4967. doi: 10.1038/s41467-018-07179-w (PMC6251895; doi:10.1038/s41467-018-07179-w)
Supplement: Supplementary file 3 — Description of Additional Supplementary Files [file 41467_2018_7179_MOESM3_ESM.docx]

**Description of Additional Supplementary Files**

File Name: Supplementary Data 1

Description: List of cellular and viral proteins detected in the proteome of HCMV infected cells. Table S1A: List of host proteins detected in the whole cell lysate time course and their relative abundances. Column H indicates if at least one acetylation site was detected on that protein in our anti-acetyl-lysine IP. Columns I-L show the average from the three replicates of the log2 intensity of the time point normalized to mock. Columns M-AA show the normalized intensity for each replicate at each time point (m, 24, 48, 72, 96). Blank values indicate that the protein was not detected (and quantified) in that sample. Columns AB-AP indicate if the protein was quantified or not in the given sample. The terms are part of the output from Protein Discoverer PD2.2 and refer to the peptides that were used for protein quantification. “High” indicates that at least one unmodified peptide from that protein was sequenced by MS/MS in that particular sample. “Peak Found” indicates that the peptides in that particular sample were found by feature matching to at least one other sample in which the peptides were confirmed by MS/MS sequencing. “Not Found” indicates that no unmodified peptides were detected for that protein in that sample. All “Not Found” cells are highlighted in green. Columns AQ-AU indicate the CV% for each time point. Columns AV-BJ indicate the number of peptides quantified for each protein in each sample. Table S1B: List of viral proteins detected in the whole cell lysate time course, and their relative abundances. Column H indicates if at least one acetylation site was detected on that protein in our anti-acetyl-lysine IP. Columns I-L show the average intensity from the three biological replicates at the indicated time point. Columns M-X show the normalized intensity for each replicate at each time point (24, 48, 72, 96). Columns Y-AJ indicate if the protein was quantified or not in the given sample. All “Not Found” cells are highlighted in green. Columns AK-AV indicate the number of peptides quantified for each protein in each sample. Table S1C: GO Biological Processes for all host proteins as determined by the Reactome plugin in Cytoscape. Processes enriched in proteins that increase in abundance, decrease in abundance, or maintain constant abundance are shown. Related terms were aggregated into broader categories for display in Figure 2a.

File Name: Supplementary Data 2

Description: List of identified cellular protein acetylations during HCMV infection. The acetylated peptides displayed in this table passed the filtering used for quantitative analysis, as indicated in the methods section. Columns I-L show the average from the three replicates of the log2 of the intensity at each infection time point normalized to mock. Columns M-P show the average (of three replicates) log2 intensity of the acetylated peptide intensity/mock normalized to the respective protein intensity/mock at each infection time point. #N/A indicates a lack of detection at the protein level. Columns Q-U show the average intensity from the three replicates for each acetylated peptide. Columns V-AJ show the intensity for each acetylated peptide at each time point in each replicate with missing values that were imputed highlighted in yellow. Columns AK-AY are the same as V-AJ except all non-detected values are indicated as NaN (preimputation). Columns AZ-BD show the CV% for each peptide.

File Name: Supplementary Data 3

Description: Subcellular localization of acetylated proteins based on prior literature knowledge. Each tab includes a list of all of the acetylated proteins (and all their respective acetylated peptides) that were assigned to each organelle (cytosol, ER, Golgi, lysosome, mitochondria, nucleus, peroxisome, and plasma membrane). The source of localization identification (Jean Beltran, et al 2016 or Uniprot) is indicated. The last column in each table indicates if the Uniprot identification aligns with the identification in Jean Beltran, et al 2016. The second to last tab is a list of acetylated proteins (and their respective acetylated peptides) that were assigned to putatively translocate during infection from their assigned localization in mock during at least one time point. All translocation assignments are from Jean Beltran, et al 2016. Cells highlighted in blue are acetylated peptides from proteins with high confidence predicted translocation from Jean Beltran, et al 2016. On each tab, log2(acetylated peptide/mock) abundance (columns E-H) and log2[(acetylated peptide/mock)/(acetylated protein/mock)] (columns I-L) are shown. #N/A in I-L indicate that no protein abundance information was collected for that protein

File Name: Supplementary Data 4

Description: : Acetylated proteins identified in both this acetylome study and Jean Beltran, et al 2016. In the upper section, organelle-resident proteins found to be acetylated at the same sites in both this study and in Jean Beltran, et al 2016 are shown. Localization assignment outside the context of infection (from Uniprot and Protein Atlas) is indicated. In the lower section, putative translocating proteins (as predicted from Jean Beltran, et al 2016) that were detected as acetylated at the same sites in both this study and in the Jean Beltran, et al 2016 dataset are shown. Proteins known from Uniprot and Protein Atlas to have multiple subcellular localizations or to translocate outside the context of infection are indicated. Additionally, the predicted localization scores from Jean Beltran, et al 2016 are shown.

File Name: Supplementary Data 5

Description: : List of viral protein acetylations identified either during HCMV infection or from enriched virions. Table S5A: The acetylated viral peptides identified in the whole cell lysate (WCL) K-Ac IPs from the infection time course are shown. Columns I-L show the average intensity from the three replicates. Blanks indicate that the peptide was not detected at that time point in any of the three replicates. Columns M-P show the average acetylated peptide intensity normalized to the average protein intensity at each infection time point. Blanks indicate that the acetylated peptide was not detected at that time point and/or that the protein abundance was not quantified at that time point. Columns Q-AB show the normalized intensity for each peptide at each time point for each replicate. Blanks indicate that the acetylated peptide was not detected at that time point in that replicate. Table S5B: This table lists the acetylated viral peptides that were identified in the virion enrichment anti-K-Ac IP.

File Name: Supplementary Data 6

Description: Lysine content in the viral proteomes of selected DNA and RNA viruses. Table S6A: Amino acid frequency analysis of 64 viral strains. The 39 strains in light blue are depicted in Figure 6b, and the remaining 25 strains shown in grey are additional HCMV or Influenza A strains. Viral strains are organized by their families and genome types (columns A-B). Total number of amino acids, number of lysine residues, and number of arginine residues are shown in columns E-G. Columns H-J show the percentage of lysine residues and the ratio of lysine/arginine residues. Table S6B: Variation in lysine percentage between different species. Table S6C and Table S6D contain the proteome analysis on subtypes of HCMV and Influenza A.
